# Impact of hydrocolloid dressings in the prevention of pressure ulcers in high-risk patients: a randomized controlled trial (PENFUP)

**DOI:** 10.1038/s41598-023-47483-0

**Published:** 2023-12-07

**Authors:** Olga L. Cortés, Victor M. Herrera, Luz D. Salazar, Yudy A. Rojas, Maribel Esparza, Alejandra Taborda, Rodolfo José Dennis

**Affiliations:** 1 Research Department, Fundación Cardioinfantil-Instituto de Cardiología, Bogotá, Colombia; 2 Nursing Department, Fundación Cardioinfantil-Instituto de Cardiología, Bogotá, Colombia; 3 Universidad Industrial de Santander, Bucaramanga, Colombia; 4 Hospitalization Services, Fundación Cardioinfantil-Instituto Cardiología, Bogotá, Colombia; 5 Nursing Department, Clínica Carlos Ardila Lulle, FOSCAL, Bucaramanga, Colombia; 6 Department of Public Health and Health Economics, Fundación Santa Fe de Bogotá University Hospital, Bogotá, Colombia

**Keywords:** Health care, Risk factors

## Abstract

It is uncertain whether hydrocolloid dressings, a more costly intervention than offering standard care with petrolatum, is superior to prevent pressure ulcers among hospitalized high-risk adults. Randomized, parallel-group, open-label, superiority trial with an active control group, blinded for investigators, event validators, and analysts (December 1, 2015 to December 12, 2017). Eligible patients were ≥ 18 years of age with intact skin judged as high-risk for skin ulcers (Braden scale), admitted to surgical or medical wards of two tertiary-level hospitals. Participants were randomized (1:1) to protection with hydrocolloid dressings or petrolatum. The primary outcome was the first occurrence of pressure ulcers (with post-injury photographs adjudicated by three judges) under intention-to-treat analysis. Based on prior cost analysis, and the available resources (assumed incidence of 6 ulcers/1000 patient-days in controls), inclusion of up to 1500 participants allowed to surpass a one-sided superiority threshold > 5% based on a target efficacy > 40% for dressings. We planned an economic analysis using a decision tree model based on the effectiveness of the study results from a perspective of the third payer of health care. After inclusion of 689 patients (69 events), the trial was stopped for futility after a planned interim analysis (conditional power < 0.1 for all scenarios if the trial was completed). Pressure ulcers had occurred in 34 (10.2%) patients in the intervention group [9.6 per 1000 patient-days] and 35 (9.9%) participants in the control group [7.9 per 1000 patient-days], HR = 1.07 [95% CI 0.67 to 1.71]. The estimated incremental cost for dressings (a dominated strategy) was USD 52.11 per patient. Using hydrocolloid dressings was found similar to petrolatum for preventing pressure ulcers among hospitalized high-risk patients. As it conveys additional costs, and in this study was unlikely to demonstrate enough superiority, this strategy did not overcome conventional skin care.

**Trial registration:** ClinicalTrials.gov identifier (NCT number): NCT02565745 registered on December 1, 2015.

## Introduction

Pressure ulcers (PU) are lesions of the skin and the subcutaneous tissues that result from continuous pressure exerted on a prominent bone (The National Pressure Ulcer Advisory Panel [NPUAP], 2019)^1^. These lesions occur more frequently in elderly patients with limitations of movement or who are immobilized for long periods and may increase their mortality risk^1, 2^. Worldwide, it is estimated that the prevalence of PU is between 5 and 15% in hospitalized patients and is considerably higher in patients admitted to intensive care units-ICU- (between 15 and 25%)^3, 4^. A systematic review and meta-analysis of the global prevalence and incidence of PU, which included cross-sectional and longitudinal studies conducted in hospitals in Asia, Australia, Europe, the Middle East, North America, and South America (surgical, medical, or ICU patients) between 2008 and 2018, reported an estimated pooled prevalence of 12.8% (95% CI 11.8–13.9), and a hospital-acquired pressure injury rate of 8.4% (95% CI 7.6–9.3)^5^. The total cost of managing a PU is estimated to be between $2.2 and 3.6 billion dollars/year. Likewise, general PU prevention strategies have shown cost savings per patient of up to US$2500 and approximately US$7276.35 in the total cost of care^6^.

The need to reduce pressure ulcers (PU) in hospitalized patients with a high risk of developing these lesions has demanded the evaluation of the effectiveness of preventive strategies^1–7^.

However, conventional preventive nursing strategies for PU have been evaluated with low certainty evidence of efficacy, and only some have cost–benefit evaluation.

Evaluated interventions are repositioning patients in their beds, protecting the skin with topical agents or with dressings^8, 9^, others are the use of foam surfaces (i.e., pillows), and strategies based on major technology, such as high-density foam mattresses and high-quality support surfaces^7, 10^. Some efficacy in preventing PU has been reported with strategies such as alternating pressure (active) air surfaces^7^ and skin protection with multi-layer silicone-adhesive polyurethane foam dressing (for the sacral area) being these last strategies the ones that have proved their efficacy in preventing PU^8, 10–15^.

The topical agents include, for example, fatty acids, olive oil, Dimethylsulfoxide (DMSO) cream, Conotrane, and Prevasone^8, 9^; and types of dressings refer to those made of polyurethane, padded dressings (hydrophobic, made of polyurethane), and hydrocolloid dressings ([HCD] made of gelatin, pectin, and carboxymethylcellulose for areas different to the face^7, 16, 17^. The insufficient evidence on their efficacy in prevention was verified in a meta-analysis of five clinical trials (n = 940) evaluating the protective effect of the combination of dressing and topical agents compared to conventional care, showing no benefit or harm in the prevention of PU (RR = 0.78, 95% CI [0.47, 1.31])^17^. The advances and clinical implementation of dressings and topical agents have been affected by the limitations on the methodology process of the studies intending to prove their efficacy in preventing PU. The consequences impact the validity of the results, making it challenging to consider in clinical decisions about care^9, 17, 18^.

As an antecedent of the present study, we performed a PENFUP-pilot study (Prevention of Pressure Ulcers by Nursing) 2015, a retrospective cohort study. It included 170 high-risk adults admitted to a preventive skin-care program in a Third-level hospital^18^. The aim was to examine the association between the preventive use of HCD and the emergence of PU in hospitalized patients. The study showed a crude hazard ratio (HR) of 1.35 [95% CI 0.58 to 3.14, p = 0.485], indicating no significant differences in PU between those exposed and those not exposed to HCD dressing. However, the uncertainty about the results attributable to the low power of this study, which is very similar to other studies reported in the scientific literature^17^, emphasized the need to structure a large randomized controlled study.

In this way, the PENFUP study was planned to assess the efficacy of hydrocolloid skin dressing (HCD) protection compared to conventional care (petrolatum or petroleum jelly [Vaseline]) for reducing the incidence of PU in hospitalized adults at high risk of developing this complication. In addition, we aim to report the time to first walk during hospitalization and estimate the cost-effectiveness of skin-dressing protection.

## Methods

### Trial design

PENFUP study was a multicenter, randomized (allocation by opaque sealed envelope), double-masked, parallel-group 1:1, controlled trial (ClinicalTrials.gov ID: NCT02565745), to evaluate the efficacy of hydrocolloid dressings and cost-effectiveness compared with the control group (conventional care using petrolatum-Vaseline^®^ brand) to prevent pressure ulcers in adult patients at risk.

Participants were allocated to the intervention group or to the conventional care prior to signing the informed consent form between January 1, 2015, and December 12, 2017, at two Third-level hospitals in Colombia at two Third-level hospitals in Colombia.

### Participants

#### Eligibility criteria

Eligible patients were adults (≥ 18 years old) admitted to the hospital within 24 h of initial consultation with intact skin (without pressure ulcers) at admission and classified as having a high [total score 10–12] or very high risk [total score ≤ 9] of PU according to the Braden scale^19^. Patients were stratified according to the reason for admission, having surgical or medical status. Patients admitted for medical conditions were included if the length of stay was going to be ≥ 24 h, and patients scheduled for surgery were eligible if their expected post-surgical duration was ≥ 4 h and would require hospitalization in an intensive care unit for ≥ 24 h. Excluded patients included those with pressure ulcers at admission time and patients with injury to the anal sphincter in admissions. Furthermore, patients whose attending physicians did not consent to participation in the study and those chronically wearing diapers were excluded.

### Settings

The study was implemented in two hospitals [each one with 300–350 beds] of fourth level of complexity. Hospital wards included hospitalization, emergency room, surgical services, and intensive care units.

### Intervention

Patients in this group from both hospitals received HCDs. These dressings have a thin layer and are translucent waterproof, composed of sodium carboxymethylcellulose gelatin. These are described as flexible, moldable, extra thin high-tech dressing, proper to protect those fragile areas exposed to greater pressure. Its duration is approximately 5–7 days; however, if his condition at the end of this time is adequate, it could not be moved, but with monitoring of his deterioration for change.

The dressings were prescribed by their physicians (in the patient´s electronic chart) based on baseline evaluation of the risk (Braden scale)^19^ of each patient and the number of body areas that would be covered during hospitalization. The size for the dressings was standardized, as well as the frequency and indications of change. A trained professional nurse in each hospital applied the dressings and changed or decided the removal time plan (every five to seven days only if their characteristics were altered by humidity or were contaminated by secretions). The skin areas selected for protection with dressings were defined according to the more prevalent areas for PU and their relations with positions (prone, supine, or lateral [right or left]) where each patient would be in for more time in bed during the length of hospitalization. Patients in a prone position received care in 10 areas, while those in a supine position received care in 11 areas, including equivalent areas on the left and right sides. Patients in one lateral position received care in six areas, as described in Table 1.

**Table 1:** Body areas defined for protection with HCD or petrolatum according to the patient´s position during hospitalization.

Supine position: posterior body plane areas protected				Prone position: frontal body plane areas protected				Lateral position: protected areas	
Site	Total	Right	Left	Site	Total	Right	Left	Site	Right/or left
Shoulders	2	√	√	Forehead	1	–		Auricular pavilion	1
Sacral	1	–		Cheekbones	2	√	√	Lower costal margin	1
Heel	2	√	√	Chin	1	–		Trochanter	1
Ankle	2	√	√	Lower costal margin	2	√	√	Ankle	1
Elbow	2	√	√	Iliac crest	2	√	√	Elbow	1
Trochanter (hip)	2	√	√	Knee	2	√	√	Knee	1
Total areas	11				10				6

### Control

The patients assigned to the control group received the conventional care or usual care of protecting the skin with petroleum, which is a semisolid combination of hydrocarbons obtained from refined petroleum; it is also known as petrolatum, white petrolatum, or soft paraffin, as well as Vaseline, which is a brand name. It is widely used in the pharmaceutical and cosmetic industry and is the excipient of many prevention methods. Petrolatum functions as an inert barrier and moisturizer, able to penetrate the upper layers of the substratum corneum. Specifically, it decreases water loss through the epidermis, and prevent kin dryness maintaining skin moisture. Further, it is easy to apply and remove; the skin can be seen through the jelly, and it is inexpensive and widely available^19^.

This intervention had to be applied without massage once by shift (once in the morning, afternoon, and once at night) by nursing staff in charge of caring the patient, in the same high-risk areas as the intervention group and was standardized in both hospitals (Table 1).

All participants in both the intervention and control group were repositioned every 2–4 h, avoiding friction, using support surfaces [pillows] to reduce pressure, and using the anti-bedsore mattress, in accordance with the same clinical practice guidelines implemented in both institutions for 10 years^7^.

### Procedures

All participants consented to have a photographic record of all areas defined in the study at the baseline and for the pressure ulcer site in case it developed. A photographic manual was developed to standardize the method of taking the photographs (metric, angle, distance, light, background, and the number of photographs) (Table 1).

Nurses of each ward evaluated the skin's integrity based on direct observation of the skin or through the HCD transparency, and observations were registered on each patient's chart. Two research nurses, one from each hospital, were trained to follow the state of the skin up of patients included in the studies. These nurses monitored the electronic medical records daily to identify new pressure ulcers (PU) reported by the nurses of each ward.

The following of the patients finished if one of these conditions was present (the first to happen: (1) the patient began to walk, or (2) was transferred or discharged to another, (3) died, or (4) developed the first pressure ulcer (First PU since the admission into the trial). Once a PU was identified and classified by the trained nurses in any group, the skin-care group of each hospital assumed the wound care. All patients were followed until discharge to include essential information related to variables and costs. The financial office provided invoices for each patient for each institution.

### Outcomes

The primary outcome was the estimated effect (crude and adjusted) of the dressings compared to petrolatum on the first pressure ulcer to appear in any of the protected areas. PU was classified as grade I to IV according to The Pressure Ulcer Advisory Panel Consensus Development Conference (NPUAP), 1989^9^. We did not use the new pressure ulcer terminology (pressure injury) and the updated stages provided by The National Pressure Ulcer Advisory Panel-NPUAP^20, 21^, given that our study started in 2015 and the national Guidelines have not been actualized^22^. The time-to-first PU was defined as the difference in time between the randomization date and the event appearance date. Furthermore, we evaluated the time of the first ambulation as an indicator of the less PU in hospitalized patients. It was defined as the time difference between the date of admission and the date in hours to which a patient initiated the first ambulation.

### Event validation

All pressure ulcers were evaluated by three experts in wound and ulcer management who adjucated the events^23, 24^. They were unaware of treatment allocation and did not know each other. The event [PU] adjudicators received instructions about the PU classification to evaluate lesions (Grade I–IV). They independently evaluated the same sets of photographs (baseline with no PU and its peer with PU) and rated the presence of PU (nominal), and classified the degree of each PU^20, 21^.

### Sample size

Considering an incidence of PU in the conventional care group of 4–6%, based on the exceeding costs of the HCD and the available resources (allowing to recruit up to 1500 patients), to demonstrate a superiority with a minimum threshold of 5%, we expected a reduction > 40% in the incidence of PUs with a power of at least 80% (one-sided alpha 2.5%).

### Randomization

After providing written informed consent, patients were randomized, using a computerized system that generated random numbers for a ratio of 1:1 to the intervention or the control group. The numbers were generated by an engineer and provided in closed-opaque envelopes to the nurse coordinator to apply to the patient once the consent form was completed. Patients were assigned to the intervention (HCD) or control group (petrolatum), and researchers, outcome adjudicators^24^, and analysts were blinded to the study-group assignments.

### Cost-effectiveness methods

A cost-effectiveness analysis was carried out based on a decision tree model from the health system's or third-party payer's perspective^25, 26^. The probabilities were taken from this multicenter "PENFUP" clinical trial. Clinical records were reviewed for the cost component of pressure ulcers, and data about care was validated in each hospital. Some rates were taken from the market according to the health services contracting policy (ISS-2001 ± 35%) in Colombian pesos for 2017. Model assumptions included a time horizon of less than one year, during the hospital stay of the medical-surgical wards and in ICU patients, the distribution of probabilities according to this trial results [probability of developing PU with HCD was 34/337, average value of 0.102, Minimum value of 0.072 and maximum value of 0.139; probability of developing PU with petrolatum was 35/352, average value of 0.099, minimum value 0.070 to maximum value 0.136] concurrent with the economic evaluation, and also the costs of pressure ulcer related to grade I and II (Table 2).

**Table 2:** Costs included in the model and probabilities.

Cost [USD] 2017	Distribution type	Average value (base case) $	Minimum value $	Maximum value $
Pressure ulcer grade I	Uniform	92.437	83.19	101.681
Pressure ulcer grade II	Uniform	132.38	119.14	145.62
Hydrocolloid dressing, HCD	Uniform	689	57.05	90.49
lubrication with petrolatum	Uniform	16.90	2.01	23.77
Day- stay in ward	Uniform	172.44	74.37	439.19
Day- stay in ICU	Uniform	101.54	91.39	1095.01
Probabilities	Evets/n			
Probability of developing ulcer with HCD (beta)	34/337	Beta	0.102	[0.072 to 0.139]
Probability of developing ulcer with petrolatum (beta)	35/352	Beta	0.099	[0.07 to 0.136]

Furthermore, it was calculated the incremental cost-effectiveness ratio (ICER). This ratio indicates the additional cost for each unwanted event avoided. To inform the health decision-making process, the ICER was compared to a cost-effectiveness threshold. The model used the threshold recommended by the WHO of up to three times the GDP per capita for Colombia, which, according to the World Bank, was USD 5805.61 (2016), USD 17,417. With the average exchange rate (TRM) of 2017 at COP 2939.63. So, the equivalent threshold was COP 51,199,052.04.

The time horizon was less than 1 year, and the cost-effectiveness threshold was three times the GDP per capita (around 50 million of COP, equivalent to 17,012.58 USD, 2017). Uncertainty was controlled with deterministic and probabilistic analyses using a Monte Carlo simulation.

### Statistical analysis

Continuous and discrete variables were summarized at baseline using means (standard deviations) and counts (percentages). Non-normally distributed continuous variables were alternatively summarized using their median and interquartile ranges. The primary efficacy analysis was based on the first occurrence of an event (PU), and we planned an intention-to-treat analysis. We estimated incidence rates (per 1000 person-days of follow-up) and 95% confidence intervals (95% CI) for each study outcome. We conducted a time-to-event analysis to contrast survival and hazard functions between groups. Survival was non-parametrically estimated using the Kaplan–Meier method, and differences between groups were evaluated using the log-rank test. We estimated hazard ratios (HRs) and 95% CI using Cox regression analysis upon testing the proportional-hazard assumption based on Schoenfeld residuals.

An interim analysis was planned after reaching the first half of the expected sample size (750 participants) or recorded events (n = 45) by an independent researcher with access to the whole database, including the codes of the study experimental arms^27^.

### Ethical considerations

Both centers obtained ethics approval before starting recruitment ([1_Comité de Ética de Investigaciones de la Fundación Cardioinfantil Instituto de Cardiología CEIC-IRB 00007736]; 2_Comité de Ética en Investigación de la Fundación Oftalmológica de Santander-FOSCAL CEI-IRB 890.205.361-4]). Each participant gave written informed consent, and we assured data confidentiality. We declare no conflict of interest related to any HCD or the petrolatum brand. The Administrative Department of Science, Technology, and Innovation financed the study- a government agency research grant.

The design of this project complied with the ethical provisions mentioned in Res. No. 008430 of 1993 is within the guidelines suggested in the Declaration of Helsinki of the World Medical Association, CIOMS, and the Belmont Report. The study was approved in two hospitals by their respective research committees (IRB Research Committee-CEIC-IRB00007736FCI; CEI-FOSCAL890.205.361-4) in Colombia. All participants gave written informed consent and we assured data confidentiality.

The proposal was classified within the research category with minimal risk since it was a clinical trial that used routinely implemented strategies in the preventive care of pressure ulcers; only on this occasion were the interventions randomly assigned, with the consent of patients and to evaluate the efficacy and actual institutional costs.

## Results

This report is based on the information submitted for a planned interim analysis, which led to decide to stop this trial earlier than anticipated. At the time, there were 689 of the 1500 expected participants (see analysis details in the “Outcomes” section).

### Data collection

**Figure 1:**
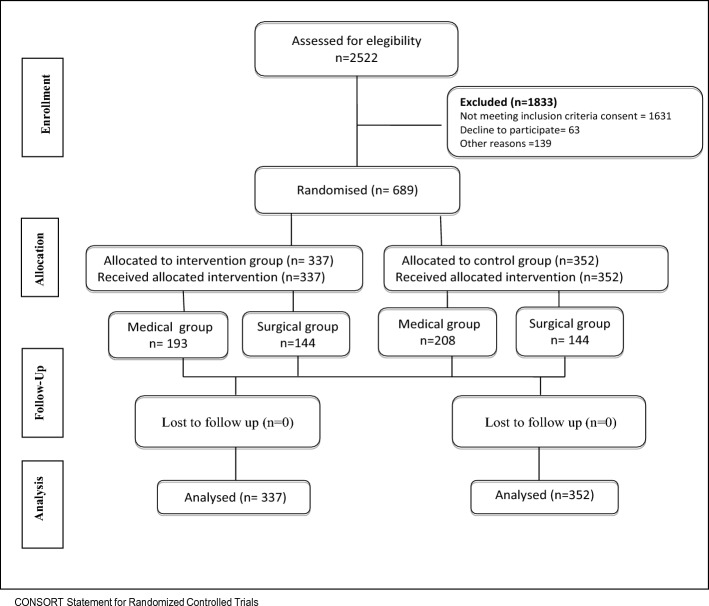
PENFUP flowchart.

A total of 2522 patients were screened. Of these patients, 1631 were not eligible, and 202 patients, although eligible, were not included for administrative reasons (n = 106), or because physicians did not authorize the patient’s participation in the study (n = 33), or the patient did not consent (n = 63) (Consort Fig. 1).

### Patients

Recruitment into the study was stopped early for futility after the interim analysis. The study included 689 patients by the time interim analysis was performed. Of these patients, 337 were assigned to receive HCD (193 in medical and 144 in surgical treatment), and 352 were assigned to receive conventional care (208 in medical and 144 in surgical treatment) (Table 3). General baseline characteristics were similar in both groups assuring a correct randomization process (see Table 3). The mean age was 62.4 years for the intervention group and 59.8 years for the control group, 52.6% were men., and 62.5% of the patients had very high risk (≤ 9), and 37.5% had high risk (10–12) for PU. Over 92.7% of the patients required to be in supine position during hospitalization due to the type of treatment or procedure required.

**Table 3:** Baseline characteristics.

Characteristics	Hydrocolloid dressing (n = 337)	Petrolatum (n = 352)
Age (year), *mean (SD)*	62.4 [21.6]	59.8 [24.9]
Male sex, *n (%)*	181 (53.7)	185 (52.6)
Recruitment Center		
Hospital 1	127 (37.7)	138 (39.2)
Hospital 2	210 (62.3)	214 (60.8)
Braden scale		
Very high risk: 9 or less	211 (62.6)	220 (62.5)
High risk: 10–12	126 (37.4)	132 (37.5)
Type of management		
Medical	193 (57.3)	208 (59.1)
Surgical	144 (42.7)	144 (40.9)
Position		
Supine	312 (92.6)	331 (94.0)
Prone	18 (5.3)	10 (2.8)
Lateral	7 (2.1)	11 (3.2)
Highest level of education completed		
Primary school	196 (59.2)	220 (63.4)
High school	106 (32.0)	100 (28.8)
Body mass index (BMI) (kg/m^2^)* mean (SD)*	25.4 (5.5)	25.3 (5.4)
In bed > 24 h pre-admission to hospital	62 (18.4)	64 (18.2)

Table 4 describes the patient´s medical antecedents, and progress along hospitalization. There were no significant differences between both groups. Medical antecedents more prevalent were Polymedication, osteoarthritis, cancer (31.5 to 27.6) and cardiovascular disease. More significant proportion of patients were admitted for medical reasons (57 to 50%), around 62 to 63% of patients had to be hospitalized in ICU and had to receive vasoconstrictor (23 to 25% and skeletal muscle relaxant treatment (33 to 32%).

**Table 4:** Comparison of patient’s characteristics on admission and reason of hospitalization between groups.

Characteristics	Hydrocolloid dressing (n = 337)	Petrolatum (n = 352)
Medical history, n (%)		
Polymedication	102 (30.3)	96 (27.3)
Osteoarthritis	112 (33.2)	109 (31.0)
Cancer	93 (27.6)	111 (31.5)
Cardiovascular	89 (26.4)	81 (23.0)
Pain	71 (21.1)	65 (18.5)
Obesity	61 (18.1)	57 (16.2)
Neurological	60 (17.0)	68 (19.3)
Diabetes	49 (14.5)	53 (15.1)
Surgical cause for hospitalization, n (%)	144 (42.7)	144 (41.7)
The surgery was performed	144 (100.0)	144 (100.0)
Surgical time (hours)	4.7 (2.0)	4.8 (1.8)
Medical cause for hospitalization, n (%)	193(57.3)	208 (58.3)
Hospital stay, treatments in ICU and complications		
Hospital stay (days), mean (SD)	8.0 (14.0)	9.0 (14.5)]
Hospitalization in ICU, n (%)	164 (48.7)	189 (53.7)
Medical- ICU	103 (62.8)	119 (63.0)
Surgical- ICU	61 (37.2)	70 (37.0)
Total time in ICU (hours)	120.0 [240.0]	131.5 [194.5]
Complications, n (%)	197 (58.5)	210 (59.7)
Medications, n (%)		
Hypnotics	81 (24.0)	94 (26.7)
Vasoconstrictors	78 (23.2)	91 (25.9)
Skeletal muscle relaxants	114 (33.8)	114 (32.4)
Medical device, n (%)		
Mechanical ventilation	106 (31.5)	121 (34.4)
In-hospital ambulation, n (%)	239 (70.9)	248 (70.5)

### Photographic records and dressings

We obtained a total of 7446 baseline photographic-records (average of 10.8 per patient) from all body areas under study at baseline of the study and 6740 photographic-records of body areas at the end of the study (average of 9.7 per patient). One thousand three hundred and forty-four (1344) dressings were used to cover 312 supine patients, 72 were used to cover 18 prone patients, and 24 were used to cover seven lateral-position patients (lateral right or left). For each patient in the supine position, we used three HCDs of 15 × 15 cm and one HCD of 20 × 25 cm (a total of 3425 HCDs); for prone patients, we used four HCDs of 15 × 15 cm (a total of 180 HCDs); and for patients in position lateral position, we used four HCDs of 15 × 15 cm (total 42 HCDs). The areas that received MC were 3633 in the supine position, 100 in the prone position, and 66 in the lateral/SIM position, the same areas covered by dressings.

### Outcomes

Twenty-five months after recruitment started, study data was submitted for the planned interim analysis. At the time, with 46% of the expected sample there were 69 validated primary outcome events (76% of the minimum expected, 34 in the experimental arm) in a total of 7959 hospitalization days. The report recommended to stop the trial for futility, based on the calculated conditional power to surpass the minimal efficacy threshold (0.0012) under the trends expected with collected data thus far.

**Figure 2:**
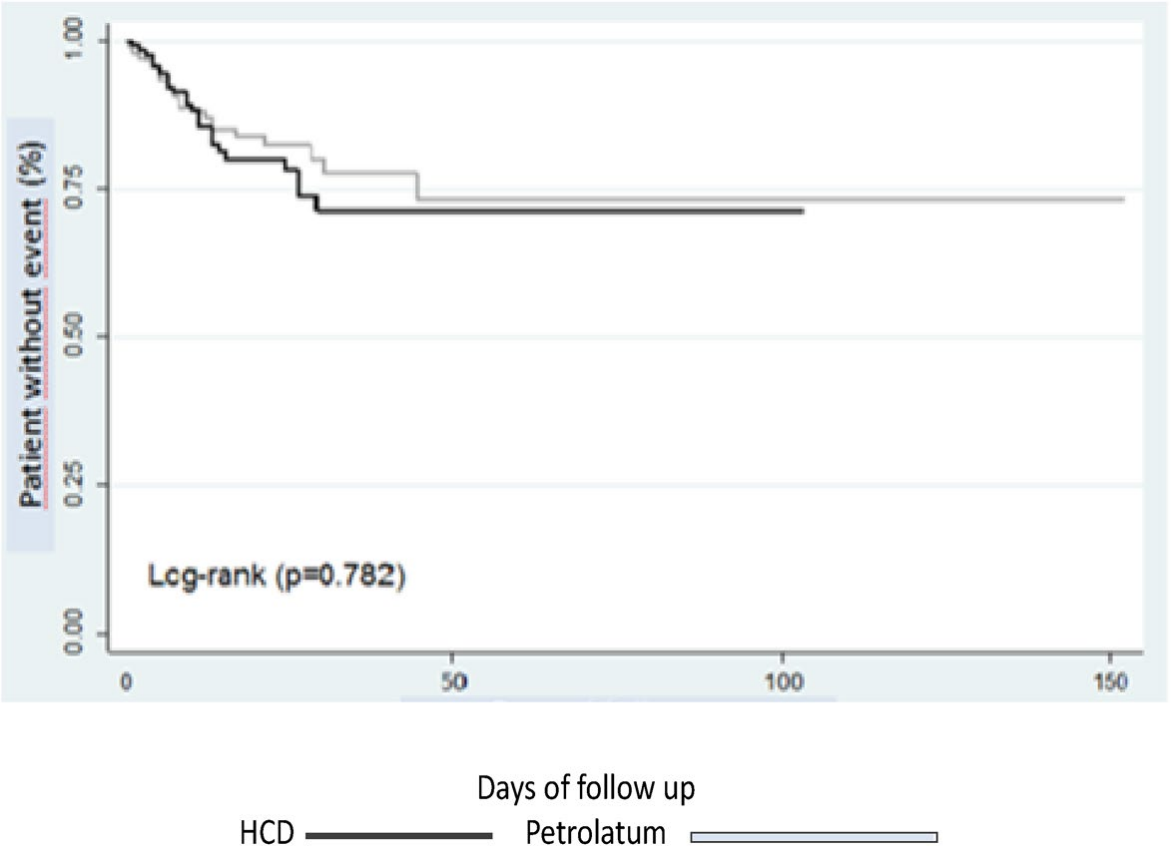
Time for the irst PU event analysis according to the study groups. Median (interquartile range) from time to event of 5.5 [3.0, 12.0] and 5.0 [3.0, 13.0] days for patients assigned to cream and dressing, respectively. Incidence rates of 7.9 and 9.6 events per 1,000 patient-days in patients assigned to cream and dressing, respectively (incidence rate ratio: 1.21, 95% CI [0.73, 2.01]).

Our findings at the closing time corresponded to an overall PU incidence rate of 8.7 per 1000 person-days: it was 9.6 for the intervention group and 7.9 for the control group per 1000 person-days (relative risk RR 1.21, 95% CI [0.74 to 2.01]). The HR raw estimate using the regression model of proportional risk was 1.07, 95% CI, [0.67 to 1.71]; the age-adjusted HR was 1.05, [95% CI 0.66, 1.69], and the BMI-adjusted was 1.07, 95% CI [0.66, 1.71], (Fig. 2). The median (interquartile range) from time to first mobilization was of 6 days [2 to 16] for control and 4 [2 to19] for intervention group (log-rank p = 0.611).

PUs were more frequently located in the sacrum (47.1% for intervention and 42.9 for control), in the right trochanter (11.8% for intervention and 8.6 for control), in the left trochanter (5.9% for intervention and 2.9% for control), and in the right heel (2.9% for Intervention and 11.4 for control group). The median time to event for PU in days was 5.5 IQR [3.0, 12.0] and 5.0 [3.0, 13.0] for patients assigned to control and the intervention, respectively.

### Economic evaluation

The alternatives have no meaningful difference in effectiveness, and only the consequences in terms of the costs of the interventions were compared. There was no significant difference in total cost [total cost of HCD 9.38140 USD [COP mean 30,262.596] and petrolatum 10.15403 USD [COP mean 32,754.933].

### Reference case

The economic evaluation was framed in terms of a cost-minimization analysis. With the model carried out, it was found that for adults over 18 years of age hospitalized, whether medical or surgical ICU patients, the use of HCD alternative or petrolatum for the prevention of pressure ulcers did show the same effectiveness (0.82) but presented an incremental cost of 52.11 USD, that is more expensive and just as effective, so the HCD it is not a cost-effective option. (Table 5). Furthermore, the deterministic sensitivity analysis 1 way probability of developing PU with HCD showed how the result of incremental cost-effectiveness for PU-prevented would change from low to high risk of developing a PU. (Table 6) If the risk of developing the event were low at 7% (for example in the first row) and the risk of petrolatum was maintained, the ICER would be 109,136 USD; and if the risk were greater than 14%, the intervention using the dressing would be a dominated alternative (more expensive and less effective).

**Table 5:** Cost effectiveness results.

Alternative	Cost USD	Incremental -cost USD	Effectiveness	Incremental effectiveness	ICER
Using HCD	80.15		0.82		
Using petrolatum	28.03	(52.11)	0.82	0.00	$-

**Table 6:** Results of the 1-way deterministic sensitivity analysis probability of developing PU with dressing.

Probability to develop PU with HD	Alternative	Cost USD	Incremental cost USD	Effectiveness	Incremental effectiveness	ICER, USD
0.072	Petrolatum	28.03		0.82		
	HCD	90.50	48.85	0.87	0.04	1091.36
0.08875	Petrolatum	28.03		0.82		
	HCD	78.77	50.73	0.84	0.02	3047.16
0.1055	Petrolatum	28.03		0.82		
	HCD	80.65	52.62	0.81	(0.01)	5008.38
0.12225	Petrolatum	28.03		0.82		
	HCD	82.53	54.50	0.79	(0.04)	1505.15
0.139	Petrolatum	28.03		0.82		
	HCD	84.42	56.38	0.76	(0.06)	924.98

**Figure 3:**
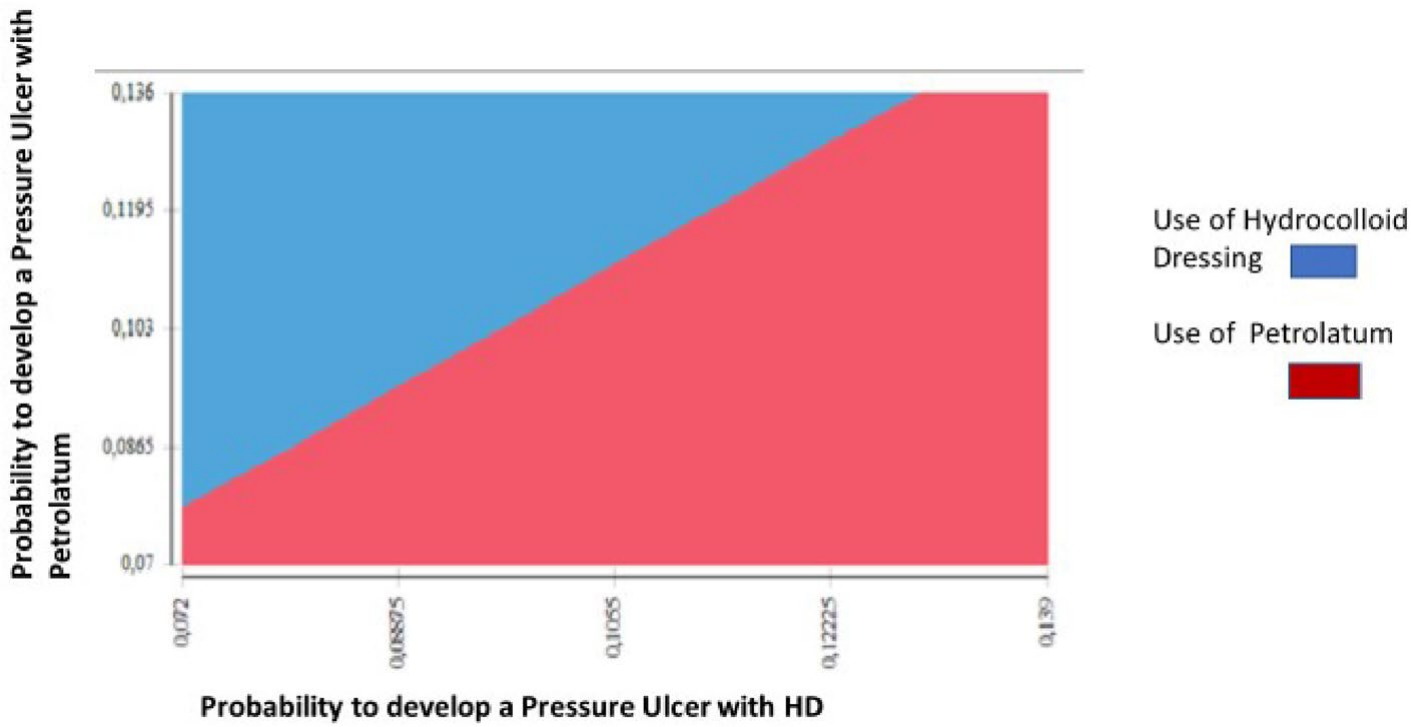
Results of the 2-way deterministic sensitivity analysis according to the probability of developing Pus with dressing and with moisturizing cream. The blue area corresponds to the dressing, which indicates that it is less likely to be cost-effective.

Finally, considering that the probability of developing PUs plays an essential role in the results of the model, a two-way multivariate analysis was performed, considering the probabilities of the event occurring. In the following graph (Fig. 3), the blue area corresponds to the dressing, which indicates that it is less likely to be cost-effective.

## Discussion

The PENFUP study evaluated the impact of preventive use of HCD compared to the administration of petrolatum in patients admitted to two tertiary hospitals for surgical or medical treatment, at high risk of PU but with intact skin. The results showed no differences in the incidence rate or the efficacy estimates between both interventions in preventing PU. However, they did present an incremental cost with the use of HCD.

Regarding the incidence of ulcers acquired in hospitalization (global of 8.7) and the prevalence of the location of the events (sacral region), our results are consistent with those reported in other studies worldwide^3, 5^. We cannot relate the results of no differences in the effect of the interventions to any of the patient factors or variables; the participants were appropriately randomized to both groups. No differences we identified on the body mass index, as it was reported in recent metanalysis^28^.

We may explain the results based on two statements. First, the capacity or ability to protect the skin by the two products under study is limited and is lower than the level of risk or potential skin damage that the patients have at the time of admission, and that may be related to multiple determinants of health. Second, the patients in both study groups received other preventive interventions in accordance with the clinical practice guidelines (CPG) established in both hospitals (RNAO) in the prevention of UP: repositioning time by schedule and use of pressure reducers (pillows). These interventions promote the mobilization and relief of body weight in some at-risk body areas.

About the first aspect, skin protective dressings have been implemented in prevention, and clinical results may vary depending on the manufacturing and properties of their materials. Bioengineering studies have described HCD (frequently used in prevention) components with little biomechanical protective efficacy. It is explained because its modulus of elasticity has a modulus ten times greater than that of the skin (in units of Pascal, kPa), which means that it exerts a more significant internal pressure, tension, and resistance, very different from the thermal characteristics of healthy skin. These items may increase the risk of injury. Bioengineering studies report that the dressings with a more excellent to lesser resemblance to the characteristics of the skin are those based on hydrogel, gels, silicones, gauze foams, and, finally, less protection is observed in hydrocolloid dressings^9^.

Concerning the use of petrolatum, the existing evidence in the evaluation of results shows some improvement in the protection of the skin as a barrier in adults hospitalized in Nursing homes in the reduction of skin tears (associated with friction and friction)^29^ and dryness in older patients who do not mobilize^11, 30, 31^. This product has shown some difference (low evidence) in preventing superficial pressure ulcers but not in the appearance of more complex ulcers (Grade II or IV)^31^, which hypothetically form from deep subcutaneous tissues, as hypothesized in other studies^16^. Thus, in PENFUP, the total number of PUs identified were Grade I and Grade II, and no PUs in stage III or IV were presented, with a more or less rapid time of appearance between four and six days after admission to the ICU.

Related to the second aspect, The studies included in this meta-analysis reported very little evidence about the use of other overlapped additional strategies for prevention during clinical trials superimposed (i.e., repositioning, use of specialized mattresses, or the use of pressure-reducing pillows) that cannot be ignored or avoided (based on no minor ethical issues), just as in our study they were administered to both groups following the usual clinical practice guidelines, probably competing in effectiveness^32^.

In addition to the above, patients included in our study were evaluated with high and very high risk with the Braden scale, indicating a high prognosis for developing a PU^33^. The level of complexity given by the alteration in health status (low cardiac output and reduced tissue perfusion), together with the administration of rescue measures such as mechanical ventilation and use of vasoconstrictors, are factors that equally and severely affected both groups. These factors ensure a transition toward a worsening state of health, affecting the entire perfusion of the body, including the skin^2^. Neither HCD nor petrolatum may have sufficient elements to prevent or reverse the formation of PUs when there is an advanced level of inadequate tissue perfusion in progress and imminent skin damage.

The evidence about the impact of the use of dressings in a hospitalized population at risk confirms the existence of uncertainty about the benefit of topical agents and dressings in preventing the incidence of PU. A meta-analysis of data from clinical trials involving more than 3000 patients, in which the effectiveness of the use of dressings, the effectiveness of the use of topical agents, and the use of combining the two types of interventions were explored, did not report evidence of the benefit of the use of HCD or topical agents in the appearance of pressure ulcers^16^. Although none of the studies reported the use of petrolatum, results about the impact of toxic agents on PU incidence were shown about fatty acids (1 study, n = 1060, RR01.28; 95% CI 0.76 to 2.17), Dimethyl sulfoxide (1 trial, n = 61; RR 1.99; 95% CI 1.1 to 3.57), Conotrane (1 trial, n = 258, RR o.74; 95% CI 0.52 to 1.07), Prevasore (1 trial, n = 120, RR 0.33; 95% CI 0.04 to 3.11) and active lotion (1 trial, n = 167, RR 0.73; 95% CI 0.45 to 1.19), with mixed efficacy results.

The use of HCD was also evaluated in this study compared to polyurethane dressings in which no differences were observed (1 trial, n = 160, RR 0.58; 95% CI 0.24 to 1.14). The results of benefit in favor of silicone dressings compared to no dressing use were evidenced in a clinical trial in this same study (n = 1247, RR0.25, 95% CI 0.16 to 0.41), especially preventing grade I and grade II PU, but not grade III, indeterminate or deeper.

The adverse events that may occur are essential in using these two interventions^34^. Although we did not observe any adverse reactions in our study, a study evaluating hydro cellular foam dressings compared to HCD (n = 80), despite not presenting PU, reported adverse events using these devices, such as peeling, skin itching, and damage to the skin due to strong adhesion^34^. Adverse events may be observed with petrolatum. However, the evidence in vitro is superior than that provided from in clinical trials^31, 35^.

In another way, studies evaluating the cost of PU showed a range of care for each PU between $20,000 and $151,700 US^25^. According to Medicare, each PU adds $43,180 US in costs for a hospital stay^25^. Economic evaluation is complex in preventing pressure ulcers, primarily because the HCD effectiveness has yet to be consistently supported by evidence. Despite this, a systematic review summarized the economic evaluations in comparative studies of interventions for preventing and treating pressure ulcers^36^. This review showed favorable results regarding cost-effectiveness in two studies (prevention) of the total analyzed with cost reductions using topic. Compared to conventional care, these studies showed that group prevention interventions are cost-effective. Group interventions included using pressure distribution or support surfaces, nutritional support, repositioning, and MC but did not include dressings. The results showed a net saving of CAN$ 3450.03 per hospitalization and an increase of 1.90 in years of quality of life. They also reported a net savings of C$55.12 per patient day, a 9.3% reduction in ulcers, and a 0.47% reduction in deaths (in 2013). These studies did not include dressings in the group interventions, most likely due to the high cost of HCDs, limited evidence of efficacy in HCD prevention, HCD research based on methodological limitations, low recommendations consistent with the GRADE system, and the need for more rigorous safety evaluations^32, 37, 38^.

If nurses or other health providers plan to use an HCD or petrolatum-based skin cream, our results suggest no proven benefit in favor of either intervention. If its use is required, it should place patients at risk in multiple possible areas and not discontinue the rest of the group interventions that can help in prevention. Decision-making about hospital implementation should allow for that HCDs show low effectiveness and high cost. Also, HCD may affect the quality of life of patients when they are removed or changed, producing pain and damage to the skin (with risk of injury). Patients could be consulted before deciding which protection strategy to use. The low level of certainty of the evidence means that additional and better-quality research is needed to advance the evidence for interventions to prevent PU.

## Importance to clinical practice

In our study, the incremental cost of HCD (intervention with low dominance) was calculated even though the two treatments evaluated did not demonstrate significant differences in effectiveness. Decision-making for their use must take into account that HCDs are devices that were initially created (recently) for wound healing and that only recently have they been promoted to be used for prevention in critically ill patients. The evidence obtained from multiple studies indicates that the materials of this product to be used in prevention require further study, and that it would be better to use, for the benefit of patients, dressings with characteristics similar to that of human skin. Petrolatum is a product created and used since 1879, and is used in the care of the skin of children and adults, on the face, in the prevention of diaper rash and in the hydration of the skin in general, it is used as part of conventional care in hospitals and nursing homes, with very low evidence of adverse effects, and with a low cost that is within the reach of the population cared for in low-resource hospitals in many parts of the world.

The two strategies lack the property of relieving the pressure that can be exerted on any part of the body in immobile patients, so their use requires the hourly implementation of other interventions that promote the relief of loads in areas under bony prominences by caregivers. These strategies, administered in groups, have shown benefit in hospitalized adult patients.

## Strengths of the study and limitations

### Strengths

The PENFUP study was a multicenter randomized controlled clinical trial. This study is unique in having administered the interventions in multiple areas of the body per patient at risk symmetrically and in the sacral area simultaneously. Likewise, it implemented the validation of ulcers using photographic records to validate ulcers and their different degrees of complexity. This study was blinded to data analysts, researchers, and pressure ulcer validators. Additionally, the cost-effectiveness study provided a key element that can help hospital health decision-makers decide whether to use any of the two interventions evaluated. Stopping the study, with a low prognosis to detect differences if the sample had been completed due to futility, provides uncertainty in the care of these patients, but can guide future research towards exploring the impact on PU prevention using other dressings with characteristics more similar to skin (i.e., silicone, hydrogel), including cost analysys.

### Limitations

Since the use of HCD is not a treatment with a clear prescription in terms of the type of patient in which it should be used, the frequency of dressing changes, and the optimal duration for each area of the body, this intervention should be used with caution in adults in critical condition.

## Conclusion

Using hydrocolloid dressings was found similar to petrolatum for preventing pressure ulcers among hospitalized high-risk patients. As it conveys additional costs, and in this study was unlikely to demonstrate enough superiority, this strategy did not overcome conventional skin care.

## Data Availability

The datasets used and/or analysed during the current study are available from the corresponding author on reasonable request.

## Abbreviations

**Hydrocolloid dressings**: Described as wafers, powders, or pastes composed of materials such as gelatin, pectin, and carboxymethyl-cellulose
**Efficacy**: Extent to which an intervention does better than harm for participants who receive the intervention
**Intention-to-treat analysis**: All patients are analysed in the groups to which they were randomised, even if they fail to complete the intervention or receive the wrong intervention
**Relative risk**: (RR) proportion of patients experiencing an outcome in the treated (or exposed) group divided by the proportion experiencing the outcome in the control group
**Hazard ratio**: The weighted relative risk over the entire study period; often reported in the context of survival analysis
